# Hats off to Modeling! Profiling Early Synthetic Dyes on Historic Woolen Samples with ATR-FTIR Spectroscopy and Multivariate Curve Resolution–Alternating Least Square Algorithm

**DOI:** 10.3390/molecules29194651

**Published:** 2024-09-30

**Authors:** Tiziana Forleo, Lorena Carla Giannossa, Anna De Juan Capdevila, Giovanni Lagioia, Annarosa Mangone

**Affiliations:** 1 Department of Chemistry, University of Bari Aldo Moro, 70126 Bari, Italy; tiziana.forleo@ispa.cnr.it (T.F.); annarosa.mangone@uniba.it (A.M.); 2 Institute of Sciences of Food Production, National Research Council (CNR), 70126 Bari, Italy; 3 Centro Interdipartimentale Laboratorio di Ricerca per la Diagnostica dei Beni Culturali, via Orabona 4, 70126 Bari, Italy; 4 Department of Chemical Engineering and Analytical Chemistry, Universitat de Barcelona, 08028 Barcelona, Spain; anna.dejuan@ub.edu; 5 Department of Economics, Management and Business, University of Bari Aldo Moro, 70126 Bari, Italy; giovanni.lagioia@uniba.it

**Keywords:** ESDs, early synthetic dyes, historic materials, ATR-FTIR spectroscopy, MCR-ALS modeling

## Abstract

This research focuses on analyzing wool samples dyed with synthetic dyes from the early 20th century. A methodology to identify and distinguish wool fibers dyed with azo, triphenylmethane, and xanthene dyes, which are no longer in use, using the ATR-FTIR spectra, is presented. Firstly, the dataset was subjected to PCA, which revealed the similarities and differences among the samples, illustrating a distribution pattern based on dye classes. MCR-ALS was employed to extract the spectral profiles of the dyed fibers, thereby enhancing the efficacy of the analytical techniques and extracting the comprehensive information from a single instrument. The combination of ATR-FTIR spectroscopy with chemometric methods, such as PCA and MCR-ALS, has proven to be an effective strategy for identifying and differentiating wool fibers dyed with early azo, triphenylmethane, and xanthene dyes. This approach has demonstrated particular effectiveness in enabling rapid analysis without requiring sampling or pretreatment. Moreover, the analysis is supported by thorough bibliographic research on these no longer used colorants. In order to maximize the potential of non-destructive spectroscopic techniques, such as ATR-FTIR, the approach used has proven to be crucial. This study underscores how chemometric techniques expand the capabilities of spectroscopy, extracting extensive information from a single instrument and aligning with the goals of cultural heritage analysis.

## 1. Introduction

**Figure 1:**
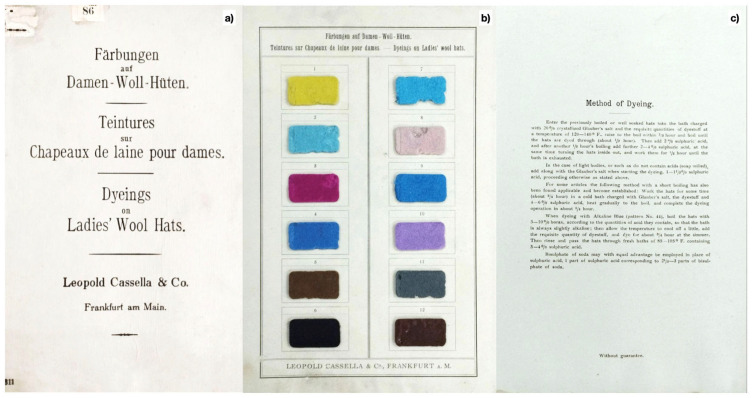
(**a**) The front cover of the pattern book; (**b**) an example page with dyed woolen pieces; (**c**) the page explaining the process of dyeing.

In this study, samples of wool dyed with early dyes from the pattern book “*Färbungen auf Damen-Wool-Hüten*/*Teintures sur Chapeaux de laine pour dames*/*Dyeings on Ladies’ Wool Hats*” published by the company Leopold Cassella & Co. (Frankfurt am Main, Germany) were analyzed. The investigated pattern book (Figure 1) is part of a massive and diversified compendium located at the Commodity Science Museum of the Department of Economics, Management, and Business Law at the University Aldo Moro of Bari (Italy) [1,2,3]. It is a sample book of synthetic dyes utilized for coloring woolen fabrics, particularly for the creation of women’s hats. It is divided into two sections. The first part provides a list of samples, the dye used, the percentage of dye used in each sample, and the etching process. The second part contains the samples presented as colored wool flaps.

The early synthetic dyes (ESDs) used to color the samples belong to three groups: the triphenylmethane dyes, the azo dyes, and the xanthene dyes.

Triphenylmethane dyes have a basic structure consisting of three aryl groups linked to a methyl group. They were among the first dyes to be developed, with fuchsine introduced in 1859 and crystal violet in 1883 [4,5].

Azo dyes are characterized by the presence of an azo group (−N=N) and linking aromatic rings. They generally contain one or more SO_3_^−^ Na^+^ groups, making them soluble in water and enabling them to bind to the surfaces of polar materials like wool, cotton, and nylon. They can also have two azo groups (diazo) or more [6,7]. The xanthene dyes, as the name suggests, are characterized by a xanthene core [6,7]. Most of the early synthetic dyes are no longer commercially available due to impractical production processes and unsatisfactory properties for textile dyeing. Consequently, they have been progressively replaced over time [7].

This study is not only informative, but it also contributes to completing the scenario for future research into the diagnosis of this sample type. As mentioned earlier, analyses in cultural heritage, especially with samples like these, necessitate methods with several characteristics, such as the following: it must be non-destructive, respecting the physical integrity of the samples; it must be fast, allowing for the analysis of similar objects or several parts of the same one; it must be universal, capable of analyzing different parts of the same sample with a single instrument, with no or minimal pre-processing; it must be versatile, offering both local and average composition of heterogeneous samples; and it must be sensitive and multi-elemental. Finding a method that satisfies all these requirements is complicated, and approaches based on the principle of “primum non nocere” remain a priority, offering the least destructive techniques that achieve the trade-off between the absence of damage and the maximum representativeness of the sample analyzed [8,9,10,11,12]. While chromatography-based techniques are the typically preferred methods of analysis, their inherently destructive nature limits their applicability in cultural heritage diagnostics [13].

Other techniques may prove useful in the study of dye tissue samples; however, they are also destructive or micro-destructive [14,15,16], and in some cases, the extraction of the dye is also required [14,16,17].

Consequently, research efforts are increasingly turning towards spectroscopic techniques [1,11,18,19].

Among spectroscopic techniques, Attenuated Total Reflectance (ATR)-FTIR spectroscopy is commonly used to determine the composition of fibers rather than colorants and their general condition [18,20]. On the other hand, UV-VIS and Raman spectroscopies are employed for colored fibers. In particular, Raman spectroscopy allows for the identification of both the type of fiber and the color of the fiber [17,18].

In addition, the use of chemometrics, which gives a considerable boost to the discrimination of colored fibers, has grown considerably in recent years [1,14,19].

Colored textiles are important samples for understanding critical issues related to scientific and technological advances, historical progress, and cultural exchanges that have taken place over centuries [21,22].

Microscopic and molecular knowledge provides information on degradation and thus guidelines for the conservation and restoration of artifacts [21,22]. In addition, it is essential to reconstruct the technological processes that led to the creation of artifacts in order to understand their capabilities, economic aspects, social significance, and cultural context [21,22]. The study of colored textiles also plays an important role in forensic science. In this field, it is not always necessary to absolutely identify the dye molecules but rather to differentiate between colored fibers in order to establish similarities or differences between trace evidence and compatible items [16,23].

In this study, ATR-FTIR was employed for sample analysis. Subsequently, the dataset underwent two chemometric approaches: Principal Component Analysis (PCA), to explore and observe similarities and differences among the samples, and Multivariate Curve Resolution–Alternating Least Square (MCR-ALS), to identify the spectral profile of the sample.

The primary objective of this study is to combine ATR-FTIR spectroscopy with PCA and MCR-ALS to identify the spectral profile of wool fibers dyed with synthetic dyes from the early 1900s. By employing ATR spectroscopy to identify colored fibers, this study aims to maximize the amount of information that can be obtained from a specific experimental technique, thereby enhancing the understanding of a sample using a single type of instrument.

## 2. Results and Discussion

This section reports the results obtained from the application of chemometric methods on the ATR-FTIR spectra to identify the spectral profile of wool fabrics dyed with different dyes (triphenylmethane, azo, xanthene). First, a PCA has been applied to explore the dataset and highlight the similarities and differences among the dyes. Afterwards, an MCR-ALS analysis allows us to understand the specific composition of the dyes.

The first part is dedicated to the study of wools colored with pure dyes with a concentration greater than 1%, as reported in the pattern book. The aims are to discriminate fabrics colored with different dyes and to identify the spectral profile of the wool fibers dyed.

The second part is dedicated to the study of the most complex dyed fabrics colored with unknown dyes and mixtures of two dyes, known and unknown (each with a concentration higher than 1%) to test the method with more complex samples.

### 2.1. Fabrics Colored with One Dye

The samples analyzed are presented in Table 1.

**Figure 2:**
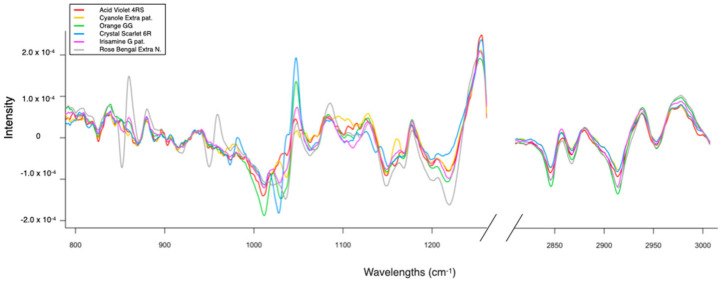
The spectra of all fabrics colored with pure dyes. The spectra are pretreated with Savitzky–Golay 1st derivative filter and are normalized.

Figure 2 shows, for each sample, the average of the three acquisitions made as the most representative spectrum. We can notice the differences between them. Each color seems to have a distinct spectral shape; just look at the difference, for example, among the GG and the CS in the range between 1000 and 1100 cm^−1^, although they have a similar structure.

#### 2.1.1. The Exploratory Analysis

**Figure 3:**
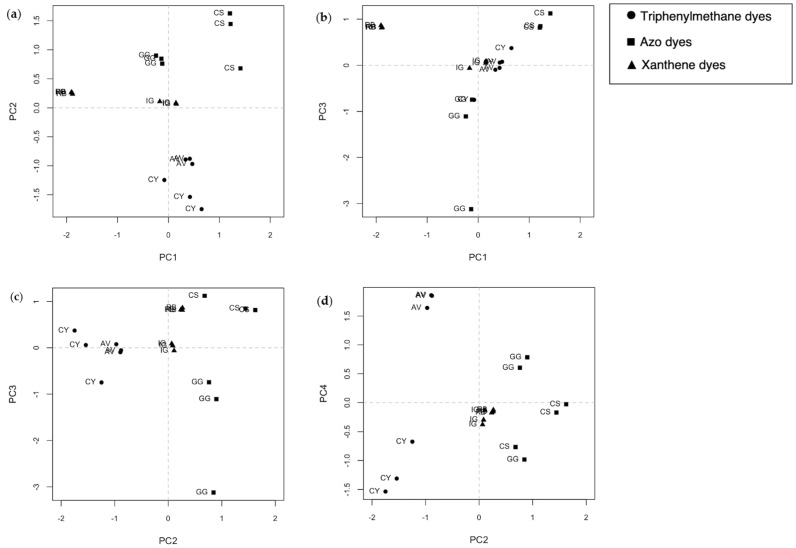
Score plots (**a**) PC1 vs. PC2; (**b**) PC1 vs. PC3; (**c**) PC2 vs. PC3; (**d**) PC2 vs. PC4. Each sample is labeled by the code presented in Table 1; the symbols indicate the a priori belonging class.

A PCA was performed as an exploratory analysis to visualize the similarities and differences between samples. The first four components describe 90.4% of the total variance (PC1 46.8%, PC2 23.7%, PC3 14.9, PC4 5.2%). From score plots in Figure 3, it can be seen that the dyed wool samples are grouped based on the type of dye and the distance between replicates, which could give us information about inhomogeneity. For example, only one replicate of GG tends to get away from the others in plots a–c. The plots also suggest that the samples can be separated by functional group according to PC2: the triphenylmethane dyes are approximately located at PC2-negative and the azo and xanthene dyes at PC2-positive.

#### 2.1.2. MCR-ALS

**Figure 4:**
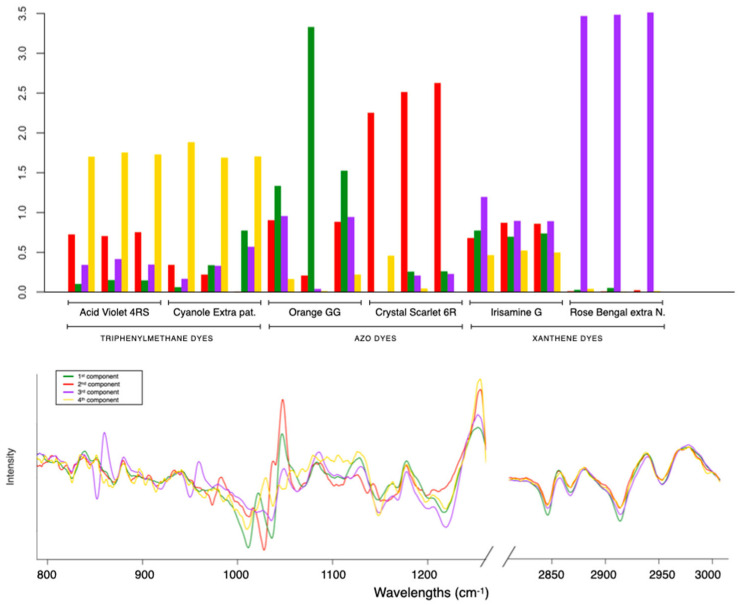
The resolved pure spectra (ST) (**bottom** plot) and the information related to the concentration profiles (C) (**top** plot).

After the exploratory analysis, the application of classical MCR-ALS was carried out. The number of components estimated by a singular value decomposition (SVD) was four. The initial estimates used were those obtained from a method based on SIMPLISMA, which was applied to the squared first-derivative corrected spectra, and MCR-ALS was performed by applying the constraint of non-negativity in concentration profiles. Moreover, with four components, a better data fit (LOF% = 11.0, r^2^ = 98.8) was achieved. Figure 4 shows the resolved pure spectra (ST) in the bottom plot, and the information related to the concentration profiles (C) in the top plot. In the latter plot, every component is represented using bars to provide an easier visualization of the composition in every sample measured.

The first and second components (in red and green, respectively) are related to the azo dyes. Both components present the pure spectra with several peaks in the range between 1030 and 1200 cm^−1^, attributed to the S-O vibration characteristic of azo dyes. The component shown in red is very dominant in the fabric dyed CS, whereas the second component is more present in the GG sample. The clear distinction between the first two components is consistent with the results of the PCA, showing the different characteristics of CS and GG (samples were far away from each other). Furthermore, the replicates of the different samples generally have a similar distribution of components (see bar distribution). In cases where the differences in intensity are greater, this could be an indication of sample inhomogeneity, as is the case with GG, which is in agreement with the PCA results. The third component (in violet) is very dominant in the xanthene dyes. The sample RB is totally described by this component. Otherwise, in the case of IG, the contributions of other components are required to define the structure.

The identified spectral features for each component can be found in the Supplementary Materials (Table S1, Refs. [31,32,33,34,35,36,37,38]). In particular, it can be noticed at 927 cm^−1^ of the in-plane bending of the fused xanthene rings, and 1234 cm^−1^ of the stretching C-O-C presented in the structure [36]. The resolved signature shows a clear presence of peaks at 955 cm^−1^, characteristic of the bond C-halogen of the RB. The fourth component (in yellow) is predominant in the triphenylmethane dyes. In this case, the high similarity of the contributions of the concentration profiles to AV and CE corresponds to the high similarity of their structures. In addition, the presence of signals attributable to the aromatic part in the region before 1000 cm^−1^ and the S-O stretching at 1028 and 1054 cm^−1^ can be observed.

### 2.2. Investigation on Known and Unknown, Pure and Mixture Dyes in Fabrics

In this section, the data matrix of fabrics colored with pure dyes (with known and unknown structures) and the binary mixture of dyes are analyzed (Table 2 added to Table 1). All the dyes considered show a concentration higher than 1%.

#### 2.2.1. The Exploratory Analysis

**Figure 5:**
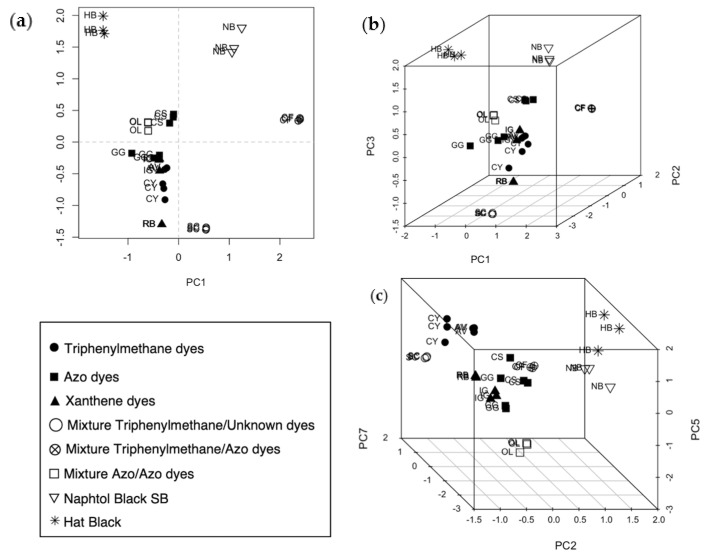
(**a**) Bidimensional score plot PC1-PC2. (**b**) Tridimensional score plot PC1-PC2-PC3. (**c**) Tridimensional score plot PC2-PC5-PC7.

A PCA was performed on the dataset. The score plots in Figure 5 show the distribution of all samples in the space of PC1-PC2 (a) and, for a better visualization, the tridimensional distribution in spaces PC1-PC2-PC3 (b) and PC2-PC5-PC7 (c).

The samples with pure dyes analyzed previously (AV, CY, GG, CS, IG, and RB) tend to group again according to functional groups. The pure dyes of unknown composition (NB and HB) are localized farther than the other samples in all the plots, but always close to the azo dyes. This behavior suggests a different structure. Moreover, they are placed near the azo and the azo–azo dyes, and this could be consistent with the hypothesis that they could be diazo dyes. When looking at the mixtures, the sample stained with Cyanole Green 6G and Solid Blue R (SC, empty circle) is far away from all the others in plots (a) and (b) but close to the sample stained with only triphenylmethanes in plot (c). This sample contains Cyanole Green 6G, which is a triphenylmethane, but it also contains Solid Blue R, which seems to belong to another class of dye, as previously stated. The mixture of azo–azo dyes, OL, is located in the same area as the azo dyes. The mixture of triphenylmethane and azo dyes, CF, is close to the azo dyes in plot (c).

#### 2.2.2. MCR-ALS

The MCR-ALS algorithm was performed on the data matrix. The number of components estimated with SVD is seven. The initial estimates used are those obtained by the SIMPLISMA method applied to the corrected squared spectra, as previously stated. The MCR-ALS algorithm is applied with the following constraint: non-negativity on the concentration profiles. Optimized MCR-ALS was performed. The results provided an LOF of 9.2% and a r^2^ of 99.2%.

**Figure 6:**
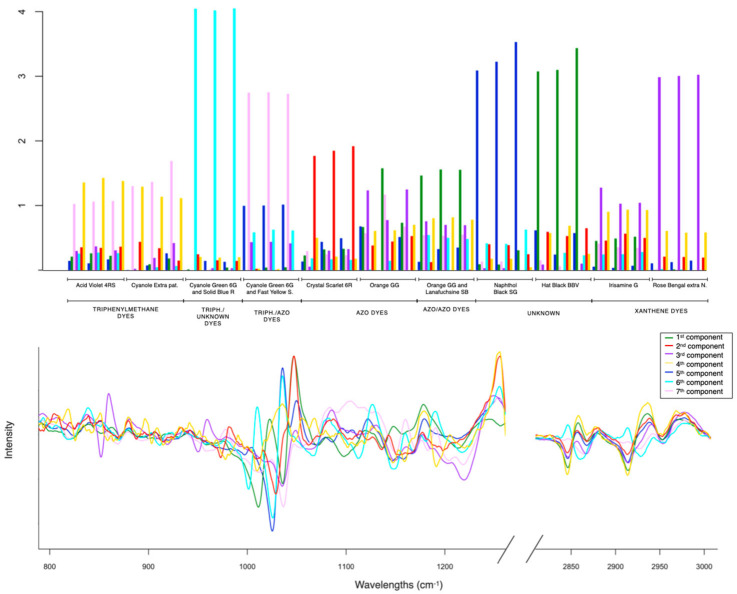
Resolved concentration profiles (C) and resolved pure spectra (ST).

Figure 6 shows the resolved concentration profiles (C) and the resolved pure spectra (ST). The interpretation of all the spectra profiles obtained is presented in Table S2 in the Supplementary Materials.

As stated before, the same components are found plus additional ones. The first component (in green) is more related to a replicate of GG and to OL and HB samples. This result, according to the PCA, suggests that the spectral profile of HB is very similar to these types of azo dyes. Once again, GG is shown as an inhomogeneous sample. As before, the second (in red) and third (in purple) components are dominant in the CS and in the sample with xanthene dyes. The third component is also present in the description of two replicates of the GG sample. The fourth component (in yellow) is related to triphenylmethane dyes. This also contributes to the description of the samples with triphenylmethane dyes and xanthene dyes. This result is in complete agreement with the PCA. The two families share the core structure of xanthene, as shown by the peak at 926 cm^−1^ (f) of the in-plane motion of the fused xanthene ring and at 1171 cm^−1^ [39], attributed to C-C-H bending.

The triphenylmethane dyes are also described by the seventh component, which is dominant in sample CF, with the mixture of triphenylmethane and azo dyes. The fifth component (in blue) is dominant in the NB and the sixth component (in cyan) is dominant in the SC. The seventh component is related to the CF, which contains a mixture of triphenylmethane and azo dyes. This component is also present in samples containing only triphenylmethane dyes.

## 3. Materials and Methods

### 3.1. The Samples

For the purposes of this study, we have focused on the wool samples with a dye concentration higher than 1%. In the first part of the evaluation, we focused on the samples shown in Table 1. Among the triphenylmethane dyes there is Acid Violet 4RS, which is described in the literature as the sodium salt of dimethylrosaniline trisulfonic acid and is also known as Red Violet 4RS [24,25,26].

Another triphenylmethane dye is Cyanole Extra Pat., which, as the name suggests, was patented by Cassella Leopoldo & Co. It is the sodium salt of m-oxydiethyldiaminophenyl-ditolyl-carbinol disulphonic acid [27,28]. Orange GG, an azo dye also known as Orange G, is formed by azo coupling between aniline and 2-(6,8-disulphonic acid)-naphthol [29]. The Crystal Scarlet 6R, also known as Crystal Ponceau 6R or New Coccine R, is another azo dye with azo groups conjugating 2-(6,8-disulphonic acid)-naphthol with naphthalene [25,30].

Among the xanthene dyes, there is Rose Bengal Extra N, which is tetra-iso-dichlorofluoresceine, also used in biological and industrial applications [25,27].

Finally, Irisamine G is a dye patented by Cassella Leopoldo & Co. and an analog of Rhodine 3G [28].

In the second part, the samples in Table 2 are added to those in Table 1.

There are three samples stained with a mixture of two dyes. The first is dyed with Solid Blue R, an unknown dye that seems to belong to the family of Induline [25] in the literature, and with Cyanole Green 6G, a triphenylmethane dye whose structure is unknown in the literature [28]. The second is dyed with Cyanole Green 6G and an azo dye, Fast Yellow S [28]. The third is dyed with two azo dyes, Orange GG and Lanafuchsine SG [28,29].

Then, there are two pure black dyes whose structure and family are unknown in the literature. Usually, they belong to the diazo family [28].

Unfortunately, in the pattern book, there is no sample of undyed wool.

### 3.2. ATR-FTIR Spectroscopy

The ATR-FTIR spectra were obtained using a Spectrum Two FTIR spectrometer (Perkin Elmer, Milan, Italy) with 32 scans, a resolution of 2 cm^−1^, and scanning from 4000 to 400 cm^−1^.

The background spectra were recorded against air. For each sample, three measurements were taken at three different points on the surface of the fabric sample.

The averaged raw FTIR-ATR spectrum of all dyed yarns, with identification of the signals attributable to wool, is presented in SM (Table S3, Figure S1, Refs. [40,41,42,43,44]).

### 3.3. Data Pre-Processing and Chemometrics

The spectra obtained by the ATR-FTIR spectroscopy were pre-processed with the Savitzky–Golay first derivative filter and normalized. The chemometrics methods applied were the PCA and the MCR.

Principal Component Analysis (PCA) is the most well-known multivariate exploratory data analysis tool. It is an unsupervised decomposition method that performs a data projection from the original high-dimensional space to a lower-dimensional one defined by a set of new variables known as principal components (PCs).

It reduces the dimensionality of the dataset and minimizes information redundancy and noise. Moreover, it enables visualization and simplifies the understanding of relationships between complex variables, thus facilitating the interpretation of the data [45,46,47].

The term Multivariate Curve Resolution (MCR) refers to a family of chemometric methods that, starting from measurements containing signals from various components (the raw data matrix D), provide a bilinear model of the individual, chemically significant contributions [48,49]:

$$D=C\;S^{T}+E$$

**Figure 7:**
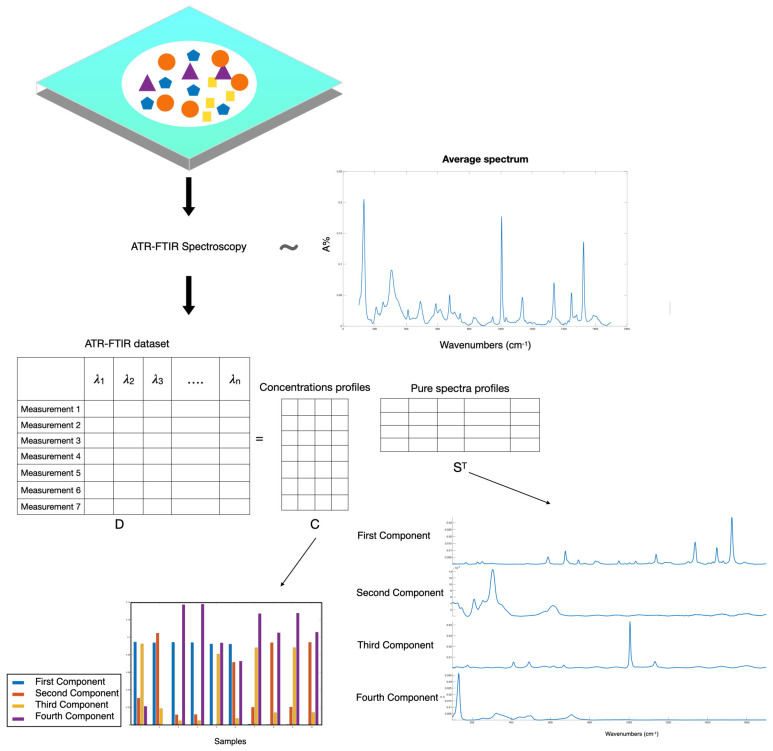
Example of the Multivariate Curve Resolution applied to a dataset of ATR-FTIR spectra.

The equation reflects the bilinear nature of spectroscopic measurements as expressed by Beer–Lambert’s law, where the variation in a dataset containing the spectra of a multicomponent system, D, can be expressed as the product of the pure spectra, S^T^, of the individual components and their respective concentration profiles, C (Figure 7).

The matrix decomposition in a bilinear model can be achieved using different algorithms [48,49]. In this work, we employ the alternating least squares algorithm (ALS). The complete method is called MCR-ALS.

One limitation of this method is that, mathematically, the original dataset can be described by different combinations of concentration and spectral profiles, a phenomenon known as ambiguity, that manifests in several ways (permutation ambiguity, intensity ambiguity, rotational ambiguity). The introduction of constraints in the MCR-ALS analysis is crucial because they incorporate both chemical and mathematical information to yield a meaningful concentration and spectral profiles while reducing or eliminating ambiguity in the MCR solutions [48,49]. These constraints encompass chemical and physical properties that are systematically presented in the component profiles within the dataset. In each iterative cycle, the calculated profiles are adjusted to comply with predetermined conditions imposed by the selected constraints. Their choice and the way they are applied are active steps of the analyst who knows the samples, the dataset, their properties, and the profiles to resolve. In this work, we apply only the non-negativity constraint, which enforces that the profiles consist of positive values. It can be applied individually to concentration profiles, pure spectra, or both.

The algorithm proceeds through several steps:

Determining the number of components in a system, which may be known or determined using methods such as PCA. Both MCR and PCA aim to describe the maximum variance present in the dataset. This is typically assessed using the Single Value Decomposition (SVD) algorithm. The analysis is further complemented by studying the noise pattern in the scores and loadings profiles. The final MCR model should give an optimal fit and chemically resolved profiles [48,49].

Generating initial estimates of C and S^T^, which can be pure spectra or concentration profiles derived from prior knowledge (e.g., known spectra of one or more components) or from systematic behavior in the concentration direction. In this work, to generate initial estimates, a purest variable selection method called SIMPLISMA is used. This method aims to select the dataset row (sample) that is most different from the others and provides initial estimates of spectra and concentration profiles.

Optimizing the spectra and concentration profiles through alterative and iterative least squares, seeking convergence while adhering to constraints. Convergence is assessed using the following parameters of lack of fit and explained variance [48,49]:

$$\%LOF=100\cdot \sqrt{\frac{\sum_{i,j}e_{ij}^{2}}{\sum_{i,j}d_{ij}^{2}}}$$

$$\%EV=100\cdot \sqrt{1-\frac{\sum_{i,j}e_{ij}^{2}}{\sum_{i,j}d_{ij}^{2}}}$$

Here, e_ij_ represents the residuals associated with the matrix decomposition using the MCR of the original dataset (d_ij_).

Optimization is deemed successful when the iteratively obtained model no longer significantly improves over consecutive iterations [48,49].

All evaluations were carried out under MATLAB (R2023a, Mathworks, Natick, MA, USA) [50] environment, using MCR-ALS 2.0 toolbox [51] and PLS toolbox (Eigenvector Research, Wenatchee, WA, USA) [52].

## 4. Conclusions

The combined use of ATR spectroscopy with chemometric methods, such as PCA and MCR-ALS, proves valuable in swiftly identifying and distinguishing wool fibers dyed with early azo, triphenylmethane, and xanthene dyes. This approach has been shown to be particularly effective for quick analysis without the need for sampling or pretreatment.

The PCA was performed as an exploratory analysis to visualize similarities and differences between samples and shows a grouping based on the types of dyes. The MCR-ALS approach allowed us to extract the spectral profile of colored fibers, maximizing the ability of the analytical techniques used and extracting the maximum information from a single instrument.

It can be confidently stated that the applied method is highly effective for analyzing simple systems, such as fabrics dyed with a single dye. However, further improvements are required to enhance its performance when applied to more complex systems, particularly those involving more dyes.

To the best of our knowledge, this is the first instance where fabric samples have been analyzed using ATR-FTIR and chemometric methodologies with the aim of extracting information beyond the fibers themselves, including the types of dyes used.

This methodology, requiring no sampling or pretreatment, streamlines the analysis process. Thorough bibliographic research supports the identification of these outdated colorants. This study highlights how chemometric techniques amplify the capabilities of spectroscopy, extracting abundant information from a single instrument and aligning with the ideal cultural heritage analysis.

## Figures and Tables

**Table 1:** Dataset A: fabrics with pure dyes with known composition.

	Code in the Pattern Book	Dye and Concentration	Identifying Name of the Sample	Family Structure	Structure	References
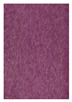	10	2% Acid Violet 4RS	AV	Tryphenylmethane	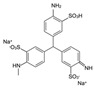	[24,25,26]
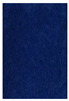	22	3% Cyanole Extra Pat.	CY	Triphenylmethane	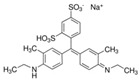	[27,28]
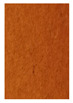	31	2% Orange GG	GG	Azo	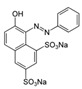	[29]
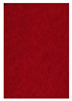	23	3% Crystal Scarlet 6R	CS	Azo	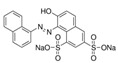	[25,30]
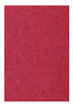	26	2% Irisamine G pat.	IG	Xanthene	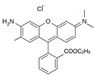	[28]
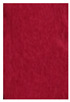	3	1.5% Rose Bengale Extra N.	RB	Xanthene	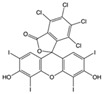	[25,28]

**Table 2:** Fabrics with a mixture of dyes or unknown samples that constitute dataset B with the samples in Table 1.

	Code in the Pattern Book	Dye and Concentration	Identifying Name of the Sample	Family Structure (Dye 1)	Structure (Dye 1)	Ref.	Family Structure (Dye 2)	Structure (Dye 2)	Ref.
Mixtures									
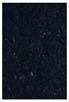	36	3.5% Solid Blue R.2% Cyanole Green 6G	SC	Unknown	Unknown	[27,30]	Tryphen	Unknown	[25,28]
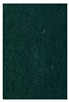	57	3% Cyanole Green 6G1.5% Fast Yellow S	CF	Tryphen	Unknown	[27,30]	Azo	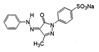	[25,28]
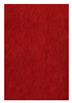	35	2.5% Orange GG1.25% Lanafuchsine SG	OL	Azo	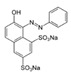	[31]	Azo	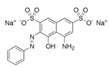	[39]
Unknown Pures									
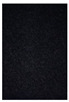	24	10% Naphthol Black SG	NB	Unknown	Unknown	[30]	-	-	
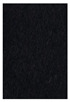	58	8% Hat Black BBV	HB	Unknown	Unknown	[30]	-	-	

## Data Availability

The data presented in this study are available on request from the corresponding author.

## Supplementary Materials

The following supporting information can be downloaded at https://www.mdpi.com/article/10.3390/molecules29194651/s1, Table S1: Interpretation of all the pure spectra obtained with MCR-ALS; Table S2: Interpretation ^a–h^ of all the pure spectra obtained with MCR-ALS for the samples presented in Table S2; Figure S1: The average raw ATR-FTIR spectrum of all dyed yarns; Table S3: Identification of the signals attributable to wool.
