# Supplementary material for: Hats off to Modeling! Profiling Early Synthetic Dyes on Historic Woolen Samples with ATR-FTIR Spectroscopy and Multivariate Curve Resolution–Alternating Least Square Algorithm
Source: Molecules. 2024 Sep 30;29(19):4651. doi: 10.3390/molecules29194651 (PMC11478277; doi:10.3390/molecules29194651)
Supplement: Supplementary file 1 [file molecules-29-04651-s001.zip › molecules-3160839-supplementary.pdf]

Table s1. Interpretation of all the pure spectra obtained with MCR-ALS.

1st Component			2nd Component			3rd Component			4th Component		
Signals (cm^{-1})	Interpretation	Ref.	Signals (cm^{-1})	Interpretation	Ref.	Signals (cm^{-1})	Interpretation	Ref.	Signals (cm^{-1})	Interpretation	Ref.
827	Ar-H out-of-plane bending	1, 4							827	Ar-H out-of-plane bending	1
						855	Ar-H out-of-plane bending	1			
874	Ar-H out-of-plane bending	1	874	Ar-H out-of-plane bending	1	874	Ar-H out-of-plane bending	1	874	Ar-H out-of-plane bending	1
			893	Ar-H out-of-plane bending	1				894	Ar-H in-plane bending	1
									907	Ar-H in-plane bending	1
927	Ar-H in-plane bending										
			929	Ar-H in-plane bending	1	929	Sym. Stretching of CCN in Ar-N(R)2	2	930	Sym. Stretching of CCN in Ar-N(R)2	6
						954	Ph-Cl				5
			982	Ar-H in-plane bending	1						
									1030	C-H in plane bending	1
			1034	S-O symm. stretching	1						
1043	S-O symm. stretching	5,7,8, 12, 13				1044	S-O symm. Stretching<br>C-H in plane bending	1, 12, 13			
									1053	sym.stertch. S-O in ion HSO3-<br>C-H in plane bending<br>NH2 rocking<br>C-OH alcol I stretching	1, 2
1076	Stretching C-O-H alcol II	1	1075	Stretching C-O-H	1	1075	Stretching C-O-H	1			
1104	S-O symm. stretching	1									
1114	S-O asymm. stretching	1	1115	S-O asymm. stretching	1	1113	S-O asymm. stretching	1			
			1121	S-O asymm. stretching	5, 12, 13	1121	S-O asymm. stretching	1, 5, 12, 13			
1172	S-O asymm. Stretching	1, 7, 13	1172	S-O asymm. Stretching	1,6	1174	C-C stretching<br>CH2 wagging<br>S-O stretching	1, 2, 4, 5	1173	C-C stretching<br>CH2 wagging<br>S-O stretching	1, 13
			1225	Stretching C-N, Stretching C-O	1, 9, 11						
1232	Stretching C-N, Stretching C-O	3, 9, 11, 13							1231	C-N stretching	1, 9, 11, 13
						1234	stretching C-N,	2, 9, 11			
2854	C-H stretching	1	2853	C-H stretching	1				2855	C-H stretching	1
2876	C-H stretching	1	2873	C-H stretching	1	2876	C-H stretching	1,2	2873	C-H stretching	1
2926	C-H stretching	1	2924	C-H stretching	1	2927	C-H stretching	1,2	2925	C-H stretching	1
2958	C-H stretching	1	2957	C-H stretching	1	2959	C-H stretching	1,2	2956	C-H stretching	1

Table s2. Interpretation [1-8] of all the pure spectra obtained with MCR-ALS for the samples presented in Table 2.

Signals (cm^{-1})

1st C.	2nd C.	3rd C.	4th C.	5th C.	6th C.	7th C.	Assignments
			825				Ar-H out-of-plane bending
826							“
				827	827	827	“
		854					“
	873						“
874					874	874	“
		875					“
			876	876			“
	890						“
					899		“
				900			“
	904					904	Stretching sym. of C-C-N arom. In Ar-N(CxHx)2
			905				“
914							“
					916		“
			921				“
925						925	
	927						Stretching sym. of C-C-N arom. In Ar-N(CxHx)2
		928					Stretching sym. of C-C-N arom. In Ar-N(CxHx)2
					932		
			933				
				935			
						954	
		955					Bending Ar-Alogen
			975				In plane C-H bending, CH3 rocking of Ar-N(CH3)2
	979						In plane C-H bending, CH3 rocking of Ar-N(CH3)2
					1006		Sym. Stretch. SO3Na, C-C-N asym bending (ring)
1018							In plane C-H bending
						1022	Sym. Stretch. SO3Na, C-C-N asym bending (ring)
				1030			S-O symm. stretching
					1032		Sym. Stretch. SO3Na, C-C-N asym bending (ring)
	1035						Sym. Stretch. SO3Na, C-C-N asym bending (ring)
1040							S-O symm. stretching
		1045					S-O symm. Stretching
							C-H in plane bending
						1052	sym.stertch. S-O in ion HSO3-, C-H in plane bending, NH2 rocking, C-OH alcol I stretching
			1071				HSO3- ion S-O symm stretching, C-H in plane bending, NH2 rocking
					1073		HSO3- ion S-O symm stretching, C-H in plane bending, NH2 rocking
	1074						Stretching C-O-H
		1075					Stretching C-O-H
						1088	
				1092			
	1112	1112					S-O asymm. stretching
1114							S-O asymm. stretching
				1115			S-O asymm. stretching
					1120		S-O asymm. Stretching, C-H in plane bending
						1121	S-O asymm. Stretching, C-H in plane bending
1143							
			1154				
					1157		
						1170	S-O asymm stretching
				1172			S-O asymm stretching
	1173						S-O asymm stretching
		1176					C-C stretching, CH2 wagging, S-O asymm stretching
					1184		C-C stretching, CH2 wagging
			1188				C-C stretching, CH2 wagging
						1205	C-C stretching, CH2 wagging
				1220			C-N stretching
	1223						C-N stretching
1225							C-N stretching
					1230	1230	C-N stretching
			1233				C-N stretching
		1235					C-N stretching
	2823						C-H stretching
						2824	“
					2840		“
	2852					2852	“
2854				2854			“
	2873						“
2874							“
		2876		2876			“
					2878		“
					2908		“
						2922	“

	2923						"
2924							"
				2925			"
		2932					"
2955	2955						"
				2958			"
		2961					"
					2962		"

Figure s1. The averaged raw FTIR-ATR spectrum of all dyed yarns.

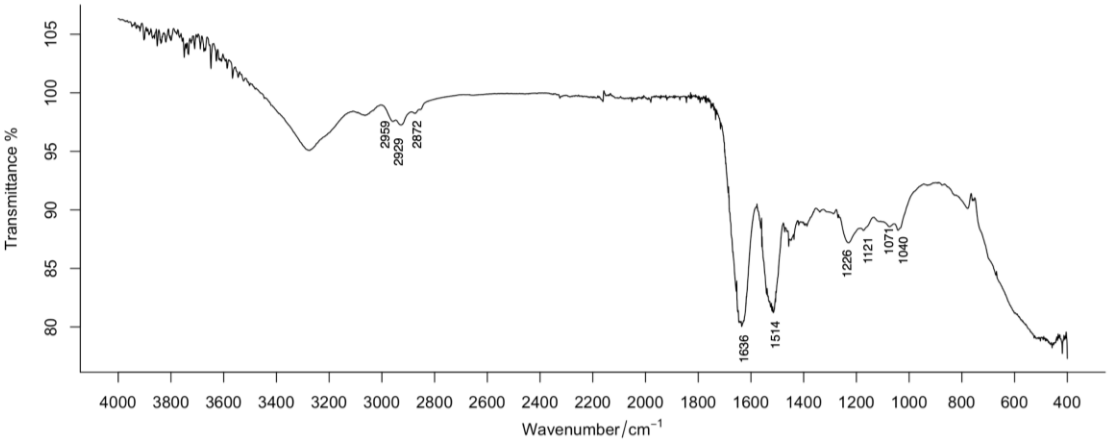

Table s3. Identification of the signals attributable to wool.

Position (cm^{-1})	Attribution
3200-3300	N-H stretching[9, 10]
2959	Stretching of C-H[10]
2929	Stretching of C-H[10]
2872	Stretching of C-H[10]
1636	Stretching C-O (amide I) [9, 10, 11, 12]
1514	N-H bending, C-N stretching (amide II) [9, 10, 11]
1226	N-H bending, C-N stretching (amide III) [9, 10, 11, 12, 13]
1121	S-O stretching of SO_2-S Cystein dioxide [12, 13]
1071	S-O Stretching of Cysteine monoxide -S-O-S[10, 12, 13]
1040	Cysteic acid - SO_3[10, 12, 13]

[1]Coates J. Interpretation of Infrared Spectra, A practical approach, Encyclopedia of Analytical Chemistry, R.A. Meyers (Ed.), pp. 10815-10837, John Wiley & Sons Ltd, Chichester, 2000

[2]Doherty, B.; Vagnini, M.; Dufourmantelle, K.; Sgamellotti, A.; Brunetti. B.; Miliani, C. A vibrational spectroscopic and principal component analysis of triarylmethane dyes by comparative laboratory and portable instrumentation, Spectrochimica Acta Part A: Molecular and Biomolecular Spectroscopy, 121, (2014), 292-305

[3]Ahmed, F., Dewani, R., Pervez, M.K., Mahboob, J., Aoomro S.A., Non-destructive FT-IR analysis of mono azo dyes, Bulgarian Chemical Communications, Vol. 48, Number 1 (pp.71-77), 2016

[4]Zubiri, I. B.; Carré A. Giving a new status to a dyes collection: a contribution to the chromotope project, Heritage, 2023, 6, 2202-2219

[5]Prati, S.; Milosevic, M.; Sciutto, G.; Bonacini, I.; Kazarin, S.G.; Mazzeo R. Analysis of trace amounts of dyes with a new enhanced sensitivity FTIR spectroscopic technique: MU-ATR (metal underlayer ATR spectroscopy), Analytica Chimica Acta, 941, 2016, 67-79

[6]Xiong, T., Zhang, Y., Donà, L., Gutiérrez,M., Möslein, A.F., Babal, A.F., Amin, N., Civalleri, B., and Tan J., ACS Applied Nano Materials 2021 4 (10), 10321-10333

[7]Al-Rubaie, L.A.R.; Mhessn, R.J. Synthesis and Characterization of Azo Dye Para Red and New derivatives

[8]Muthukumar, M.; Thalamadai, Karuppiah, M.; Bhaskar Raju G. Electrochemical removal of CI Acid orange 10 from aqueous solutions, Separation and Purification Technology, Volume 55, Issue 2,2007, Pages 198-205, ISSN 1383-5866
[9]Jiang, Z.; Li, W.; Wang, Y.; Wang, Q.; Second-Order Derivation Fourier Transform Infrared Spectral Analysis of Regenerated Wool Keratin Structural Changes. AATCC Journal of Research. 2022;9(1):43-48. doi:10.1177/23305517211060778
[10]ATR-FT-IR spectrum of wool https://spectra.chem.ut.ee/textile-fibres/wool/ (last access on 17 sept 2024)
[11]Geba, M.; Lisa, G.; Ursescu, C.M.; Olaru, A.; Spiridon, I.; Leon, A.L.; Stanculescu, I.; Gamma irradiation of protein-based textiles for historical collections decontamination, J.Term. Anal. Calorim., 2014, 118:977-985
[12]Kan, C. W., Chan, K., & Yuen, C. W. M. (2004). Surface characterization of Low Temperature Plasma treated wool fiber -The effect of the nature of gas. Fibers and Polymers, 5(1), 52-58. DOI: 10.1007/BF02875495
[13]Kissi, N.; Curran, K.; Vlachou-Mogire, C.; et al. Developing a non-invasive tool to assess the impact of oxidation on the structural integrity of historic wool in Tudor tapestries. Herit Sci 5, 49 (2017). https://doi.org/10.1186/s40494-017-0162-1
